# A preliminary study on the association between prognostic nutritional index and neutrophil-to-lymphocyte ratio with nutritional status and inflammation in febrile children's susceptibility to seizures

**DOI:** 10.1590/1806-9282.20240166

**Published:** 2024-07-19

**Authors:** İpek Dokurel Çetin, Orkun Çetin

**Affiliations:** 1 Balıkesir University, Faculty of Medical, Department of Pediatrics, Division of Pediatric Neurology – Balıkesir, Turkey.; 2 Balıkesir University, Faculty of Medical, Department of Gynecology and Obstetrics, Division of Perinatology – Balıkesir, Turkey.

**Keywords:** Prognostic nutritional index, Neutrophil, lymphocyte, Febrile seizure, Children

## Abstract

**OBJECTIVE:**

The aim of this study was to evaluate the association between nutritional status, inflammation, and susceptibility to seizures in febrile children.

**METHODS:**

This observational single-center study was carried out from January 2020 to December 2023 with 324 children aged 6 months and 6 years; 106 were diagnosed with febrile seizure, 108 were febrile children, and 110 were healthy controls. The prognostic nutritional index and neutrophil-to-lymphocyte ratio were calculated, and the cutoff threshold was established through receiver operating characteristics. The study utilized correlation and univariate–multivariate logistic regression analysis. The comparison between simple and complex febrile seizure was conducted to analyze differences.

**RESULTS:**

The optimal cutoff values were identified as 61.25 for prognostic nutritional index and 1.04 for neutrophil-to-lymphocyte ratio. Our findings showed a significant negative association between febrile seizure and platelet count, high C-reactive protein, and high ferritin levels. Additionally, the febrile seizure group showed a significant positive correlation with high neutrophil-to-lymphocyte ratio values (≥1.04) and body temperature (≥38). Our findings revealed that high neutrophil-to-lymphocyte ratio, high C-reactive protein, and age less than 18 months were independently associated with seizure susceptibility in febrile children.

**CONCLUSION:**

High neutrophil-to-lymphocyte ratio values and low prognostic nutritional index scores may serve as novel surrogate independent factors for seizure susceptibility in febrile children. Febrile children who are less than 18 months old are more prone to experience seizures than older febrile children. Moreover, there was a correlation between febrile seizures and elevated C-reactive protein levels and neutrophil-to-lymphocyte ratio values.

## INTRODUCTION

Febrile seizures (FSs) are the result of an immature brain reacting to a high body temperature. Simple FSs are considered harmless, and there is no evidence indicating an increased risk of death, neurodevelopmental problems, or epilepsy compared to the general population^1^. However, recent studies have connected complex FSs to sudden unexpected death in epilepsy, brain injury, and febrile status epilepticus^2^.

Malnutrition makes a child more susceptible to infections that result in fever, which causes seizures in susceptible children. In opposition to the body of research concerning the correlation between malnutrition and other diseases, there is a scarcity of studies investigating the nutritional status of children with FS. Heightened levels of vitamin D and B12 in the serum of febrile children (FC) can serve as a prophylactic marker against FS^3^. Various studies have corroborated that iron deficiency is also a risk factor for FS^4,5^.

Inflammation is a critical factor in the progression of spontaneous seizures^6^. Elevated neutrophil-to-lymphocyte ratio (NLR) has been noted in inflammatory conditions such as COVID-19 infection^7^, gastrointestinal diseases^8^, and thyroiditis^9^. Previous research has focused on the relationship between inexpensive and readily available blood biomarkers, such as the NLR, red blood cell distribution width (RDW), and platelet (PLT) count, in FSs^10^.

Prognostic nutritional index (PNI) was independently linked to the presence and severity of neonatal sepsis in a negative manner^11^. Additionally, PNI has been linked with inflammatory conditions such as type 2 diabetes mellitus and its complications^12^. There is also evidence suggesting a connection between PNI and patient outcomes^13^. Despite the recognized prognostic significance of PNI in the pediatric population, it is surprising that no investigation has scrutinized the correlation between FS and measures of PNI. Furthermore, the relationship between NLR and PNI scores and susceptibility to FS remains unexplored in existing literature.

We aimed to investigate the association of NLR, PNI, and the related factors with seizure susceptibility in FC.

## METHODS

This retrospective observational study, conducted in a tertiary hospital from January 2020 to December 2023, adhered to ethical principles outlined in the Declaration of Helsinki and was approved by the institutional ethics committee (November 22, 2023; number: 2023/174), and written informed consent was obtained from the participant.

Children aged 6 months to 6 years who manifest FSs are defined as displaying convulsions coinciding with a body temperature of 38°C or above, without any evidence of central nervous system infections or other causative factors for seizures. Furthermore, the participants were categorized as simple and complex FS in accordance with the American Academy of Pediatrics guidelines^14^. Simple FS lasts no more than 15 min, is generalized, and occurs only once within 24 h. Complex FS lasts longer than 15 min, has a focal origin, or occurs multiple times within 24 h. The FC group comprised children of the same age and gender who experienced fever due to acute infections but did not have seizures. Fever causes of FS and FC were classified as upper respiratory tract infection (URTI), lower respiratory tract infection (LRTI), acute gastroenteritis (AGE), urinary tract infection (UTI), and cellulitis. The final group is made up of healthy children (HC) who are brought to the hospital for routine health checks without presenting any complaints. These children are age- and sex-matched with the other groups and do not have seizures or fever.

Children with afebrile seizures, born before 37 gestational weeks, neurodevelopmental problems, metabolic abnormalities, central nervous system infections, diagnosed with other hematological problems, on nutritional supplements, and chronic systemic disease were excluded.

A total of 324 children's medical records were evaluated using a form: age, sex, body mass index (weight/height^2^), tests [for hemoglobin, RDW, white blood cells, lymphocytes, neutrophils, platelet PLT counts, C-reactive protein (CRP), vitamin B12, D, ferritin, and albumin]. The PNI is calculated as 10 × serum albumin (g/L) +5 × lymphocyte (10^3^ cells/μL), and NLR is calculated by dividing the percentage values of neutrophils and lymphocytes.

Statistical analyses were performed using SPSS for Windows 25.0. The test of normality was investigated using the Kolmogorov-Smirnov test. The Kruskal-Wallis tests were conducted to compare these parameters among the participants (FS, FC, and HC). Mann-Whitney U test was used for pairwise difference significance, with Bonferroni correction for multiple comparisons. The cutoff value was accessed by receiver operating characteristic (ROC) analysis. Correlations were determined by the Spearman test. Factors from univariate analyses were used for multivariate analysis via logistic regression. Differences were considered significant at p<0.05, with a coefficient interval of 95%.

## RESULTS

A total of 324 participants, 106 (32.7%) were FS, 108 (33.3%) were FC, and 110 (34%) were HC. Female gender was similar among FS (47, 44.3%), FC (54, 50%), and HC (56, 50.9%) (p=0.582). The body mass index exhibited no significant difference among FS, FC, and HC (p=0.475). The fever etiology did not differ between the FS and FC in terms of URTI, LRTI, UTI, AGE, and cellulitis (47.2 vs. 42.6%, 20.8 vs. 21.3%, 5.7 vs. 13%, 15.1 vs. 15.7%, and 11.3 vs. 7.4%, respectively; p=0.742). The difference in the age distribution among groups was similar; FS (3±1.3), FC (3.4±1.6), and HC (3±1.5) (p=0.086). In terms of age, there were no significant variations among the three groups, with FS (3±1.3), FC (3.4±1.6), and HC (3±1.5) (p=0.086).

When the nutritional status was assessed, it was found that there were no significant differences in terms of vitamin D and B12, hemoglobin, and RDW (Table 1). The analysis suggested a significant difference among groups in terms of ferritin, CRP, PNI, NLR, and PLT. Pairwise comparison revealed that the PLT of the FS was significantly higher than in the FC and HC groups (p=0.003 and 0.004 vs. 0.867). The HC's PNI and NLR were assessed through pairwise comparison and significantly higher than those of the FS and FC [p=0.002, 0.015 vs. 0.620, and p<0.001, 0.006 vs. 0.032, respectively].

**Table 1:** Comparison of biochemical and complete blood count parameters of groups in the study.

Variables	Febrile seizure n=106	Febrile children	Healthy children	p-value
Mean ± SD		n=108	n=110	
Vitamin D (ng/mL)	24±10.4	25.8±12.1	27.2±11.7	0.138^a^
Vitamin B12 (pg/mL)	346.5±172.7	365.4±193.7	377.6±196.1	0.569^a^
Ferritin (**μ**g/L)	33.7±25.7	31.2±22	26.5±27.6	**0.006** a
C-reactive protein (mg/L)	32.2±33.1	18.7±25.4	–	**<0.001** b
Body temperature (°C)	39.3±1	39±1.1	–	**0.029** b
Hemoglobulin (g/dL)	11.7±1	11.6±1.5	11.8±1.2	0.576^a^
Prognostic nutritional index	61.05±9.9	62.2±10	65.3±10.8	**0.006** a
Neutrophil-to-lymphocyte ratio	2±2.1	1.5±2.2	1.4±2.8	**<0.001** a
White blood cell	10.1±4.4	9.8±3.7	9.6±3.2	0.888^a^
Platelet count (10^9^/L)	323.1±116.2	357.5±100	358.6±107.2	**0.004** a
Red cell distribution width (%)	14.7±1.9	14.9±1.7	14.5±1.4	0.290^a^

a Kruskal-Wallis test

b Student's t-test.

Statistically significant p-values are denoted in bold.

The ROC curve analysis calculated the cutoff values of PNI and NLR to be 61.25 (AUC=0.570, p=0.041) and 1.04 (AUC=0.633, p<0.001), respectively.

Age, gender, body temperature, duration of fever prior to seizure, recurrence of FS, and positive family history of FS did not significantly differ between simple and complex FS (Table 2). The complex FS group showed a significantly longer duration of FS (p<0.001). The simple FS showed significantly higher PNI, whereas the complex FS had significantly higher CRP (p=0.001 and 0.001, respectively). No significant differences were found in NLR, RDW, or PLT between the simple and complex FS. Among complex FS, a larger proportion of children were observed to have low nutritional status (PNI≤61.25) and high inflammatory response (NLR≥1.04) (p=0.002 and 0.071, respectively). Among the 106 cases of FS, 49.1% were characterized by a single occurrence, while the remaining 50.9% (n=54) were marked by recurrent episodes.

**Table 2:** Biochemical and clinical parameters in children with simple versus complex febrile seizures.

Variables	Simple febrile seizure n=67	Complex febrile seizure n=39	p-value
Age (years)*	3±1.3	3±1.5	0.672
Gender** female	26 (38.8)	21 (53.8)	0.135
Prognostic nutritional index*	63.1± 9.4	57.6± 9.8	**0.001**
Neutrophil-to-lymphocyte ratio*	1.8±2	2.3±2.3	0.142
Ferritin (μg/L)*	33.5±27.3	34.2±22.8	0.564
Platelet count (10^9^/L)*	338.2±126.9	297±90.8	0.206
Red cell distribution width (%)*	14.9±1.9	14.4±1.7	0.21
C-reactive protein (mg/L)*	12.6±16.9	29.3±33.1	**0.001**
Vitamin D (ng/mL)*	24.8±10	22.6±11	0.175
Fever duration before seizure (min)*	51.2±70.8	54.3±79.2	0.471
Body temperature (°C)*	39.3±1.03	39.2±1	0.722
Duration of febrile seizure*	3.03±1.6	15.1±11.2	**<0.001**
Times of febrile seizure recurrences*	2.2±1.8	2.2±1.6	0.673
Positive family history of febrile seizure**	35 (52.2)	21 (53.8)	0.874
Children with low PNI**	29 (43.3)	29 (74.4)	**0.002**
Children with high NLR**	38 (56.7)	29 (74.4)	0.071

* Mean±SD

** Number (%),

Mann-Whitney U test was used. Statistically significant p-values are denoted in bold.

The study examined FS and FC by correlation analysis to determine the relationship between clinical characteristics, lab tests, low PNI scores, and NLR. There was a significant negative correlation in FS between high NLR and age >18 months (r=0.216, p<0.001), PLT (r=0.116, p=0.037), and ferritin (r=0.212, p<0.001). High NLR is positively correlated with low PNI (r=0.425, p<0.001). Low PNI was significantly negatively correlated with age >18 months (r=0.207, p<0.001), PLT (r=0.254, p<0.001), and ferritin (r=0.124, p=0.026) in FS. The FS was significantly negatively correlated with PLT (r=0.185, p=0.001), elevated CRP (r=0.329, p<0.001), and ferritin (r=0.149, p=0.007). On the other hand, FS was significantly positively correlated with high NLR (r=0.245, p<0.001) and elevated body temperature (r=0.135, p=0.049).

The study utilized both univariate and multivariate logistic regression methodologies to evaluate the factors contributing to the susceptibility of FC to seizures (Table 3). Age was categorized into two specific groups: children below and above 18 months of age, in accordance with a previous study. The multivariate analysis revealed that age below 18 months (p=0.039), high CRP (p<0.001), and high NLR (p=0.004) were independent factors related to seizure susceptibility in FC.

**Table 3:** Univariate and multivariate logistic regression analysis to determine risk factors for susceptibility to seizure in febrile children.

Variables		Univariate analysis			Multivariate analysis		
		OR	95%CI	p-value	OR	95%CI	p-value
Age		1.1	0.69–1.8	0.670	2.03	1.04–3.95	**0.039**
	>18 months						
	≤18 months						
Gender		1.3	0.8–2.04	0.302	1.3	0.7–2.3	0.453
	Female						
	Male						
Ferritin value≥11 μg/L		2.6	1.3–5.5	0.009	0.7	0.3–1.8	0.434
	No						
	Yes						
PNI score≤61.25		0.12	0.9–2.3	0.117	1.5	0.7–3.1	0.240
	Yes						
	No						
NLR value≥1.04		2.9	1.8–4.7	<0.001	2.8	1.4–5.6	**0.004**
	No						
	Yes						
Platelet count (×10^9^/L)		1	0.9–1	0.007	1	0.9–1	0.058
Body temperature≥38°C		2.1	1–45	0.052	2.3	1–5.4	0.059
	No						
	Yes						
C-reactive protein≥5 mg/L		0.16	0.07–0.35	<0.001	7.3	3–17.4	**<0.001**
	Yes						
	No						

SD: standard deviation; OR: odds ratio; CI: confidence interval. Statistically significant p-values are denoted in bold.

## DISCUSSION

To the best of our knowledge, this is the first study to explore the relationship between PNI and NLR in FC and evaluate their susceptibility to FSs. We found a significant difference in HC's PNI and NLR compared to FS and FC. The FS group demonstrated significantly higher body temperature, serum ferritin, and CRP, along with a marked decrease in PLTs. FS patients had significantly lower PNI (≤61.25) compared to HC and FC. In addition, the relationship between PNI and FS subtypes has been investigated in this article for the first time. Our study demonstrates that patients with complex FS exhibit significantly lower PNI, higher NLR, and higher CRP compared to those with simple FS. Our research highlights the significance of addressing both low nutritional status and high inflammation in FC, as they may play a key role in the mechanism of FS.

The nutritional well-being and prognostic potential of FS children have been understudied. Kumari et al. reported iron deficiency as one of the risk factors for first-episode simple FS^4^. Our findings showed that FS has significantly higher serum ferritin than other groups, and those with complex FS have even higher levels than those with simple FS. Since ferritin is an acute-phase reactant, it rises in nonspecific inflammatory conditions such as FS and FC groups. We tried to explain this discrepancy with previous studies by examining ferritin levels of HC. Vitamin D has a beneficial effect on the brain in epilepsy, both by transmitting signals between nerve cells and by protecting the central nervous system. Bhat et al. showed that vitamin D deficiency is associated significantly with simple FS, and their recurrence is negatively correlated with low 25OH vitamin D^15^. Çığrı et al. proposed that high vitamin D and B12 levels prevent the development of FS in children^3^. Deficiency in vitamin B12 could potentially lower the threshold for seizures. Surprisingly, no significant differences were found in vitamin D or B12 levels between groups in the present study. These intriguing results could be related to the dietary practices of native children. Regularly consuming animal-based foods from our land may have boosted their vitamin D and B12 levels. To fully understand the potential benefits of vitamin B12 in raising the seizure threshold and vitamin D in reducing inflammation, further studies with larger sample sizes on FSs are warranted.

A reliable way to assess nutritional and immunological well-being is through the PNI. Elevated PNI levels are strongly related to positive health outcomes, as they reflect better functioning in these essential areas. In children, the PNI was used to predict coronary artery involvement and intravenous immunoglobulin resistance in Kawasaki disease^16-18^, the outcome of human parainfluenza virus-induced pneumonia^19^, the prognosis of chronic kidney disease^20^, trace the response to treatment in pediatric leukemia^21^, and as a predictor for severity of neonatal sepsis^11^. This study confirms that the PNI differed significantly between groups. Compared to other children, those with lower PNI scores were linked to FSs. We posit that the relationship between poor dietary habits and potential impaired management of inflammation is the contributing factor in the manifestation of FS.

The NLR is often relied on as a valuable tool to predict the prognosis of infectious diseases through monitoring systemic inflammation in children^22^. High levels of NLR are strongly linked to negative outcomes, so it is advisable to consider NLR as an unfavorable prognostic factor. Aside from assessing infectious diseases in children, the NLR is also utilized to predict the prognosis of autism^23^, surgical site infection^24^, asthma diagnosis and severity^25,26^, and attention-deficit/hyperactivity disorder^27^. This study shows that NLR is strongly associated with seizure susceptibility in FC. There was a clear correlation between high NLR and FSs. This finding raises intriguing questions regarding the nature of the relationship between seizure burden and inflammation. Unfortunately, these findings are rather difficult to interpret due to the retrospective design of the study.

Immature neurons tend to synchronize and generate seizures. We found that age <18 months increased the risk of seizures twofold in FC. Another surprising variable that was found to be significantly associated with complex and simple FS was low PNI. This finding might indicate that the existence of nutritional disparities among individuals may be the underlying cause for the development of FS subtypes. The present results are significant in at least two major respects. First, it is crucial to recognize the wide-reaching advantages of a properly nourished infant, extending beyond just the expected benefits. This responsibility should be upheld by all social policies. Second, proper nutrition can help alleviate the impairments caused by inflammation in an immature brain network.

There are a number of limitations to our study, such as the lack of data on feeding techniques and daily protein intake. The study's relatively small sample size highlights the need for future studies to have larger sample sizes, a prospective design, and a longer duration of follow-up. This was a retrospective single-center study, and the research data cannot be generalized. The PNI and NLR were only calculated at admission, so continuous monitoring of the PNI and NLR might provide more significant insights into the seizure susceptibility of FC.

## CONCLUSION

Given the child's infection-related nonspecific inflammation, it would be prudent to take precautionary measures against FSs by providing appropriate nutrition. Our findings indicate that FC older than 18 months are more likely to experience seizures. FC with elevated CRP levels are more susceptible to seizures than those with normal CRP levels. Additionally, compared to children with normal NLR levels, FC with elevated NLR levels are more prone to experiencing seizures.

## ETHICAL APPROVAL

All the procedures followed by the authors were in accordance with the ethical standards of the responsible committee on human experimentation (institutional or regional) and with the Helsinki Declaration of 1975, as revised in 1983. The privacy rights of our patients were always observed. This study was approved by the Institutional Ethical Committee of Balikesir University (November 22, 2023; number: 2023/174). The groups' caregivers agreed to participate in the study by signing the relative informed consent form.
